# The Role of *Orange Gene* in Carotenoid Accumulation: Manipulating Chromoplasts Toward a Colored Future

**DOI:** 10.3389/fpls.2019.01235

**Published:** 2019-10-04

**Authors:** Claudia E. Osorio

**Affiliations:** Agriaquaculture Nutritional Genomic Center, CGNA, Temuco, Chile

**Keywords:** carotenoid accumulation, *Orange* gene, *OR*, Plastid, Orange protein, protein interaction, phytoene synthase

## Abstract

Carotenoids are isoprenoid pigments synthesized in plants, algae, and photosynthetic bacteria and fungus. Their role is essential in light capture, photoprotection, pollinator attraction, and phytohormone production. Furthermore, they can regulate plant development when they are processed as small signaling molecules. Due to their importance for human health, as promoters of the immune system and antioxidant activity, carotenoids have been used in the pharmaceutical, food, and nutraceutical industries. Regulation of carotenoid synthesis and accumulation has been extensively studied. Excellent work has been done unraveling the mode of action of phytoene synthase (PSY), a rate-limiting enzyme of carotenoid biosynthesis pathway, in model species and staple crops. Lately, interest has been turned to Orange protein and its interaction with PSY during carotenoid biosynthesis. Discovered as a dominant mutation in *Brassica oleracea*, Orange protein regulates carotenoid accumulation by posttranscriptionally regulating PSY, promoting the formation of carotenoid-sequestering structures, and also preventing carotenoid degradation. Furthermore, Orange protein contributes to homeostasis regulation, improving plant tolerance to abiotic stress. In this mini review, the focus is made on recent evidence that elucidates Orange protein mode of action and expression in different plant species. Additionally, strategies are proposed to modify *Orange* gene by utilization of genome editing techniques. A better understanding of carotenoid biosynthesis and accumulation will lead to a positive impact on the development of healthy food for a growing population.

Carotenoids are isoprenoid pigments, synthesized in all photosynthetic organisms, such as bacteria, algae, plants, and some fungi (Howitt and Pogson, 2006; Yahia et al., 2017; Young and Pallett, 2017). In higher plants, carotenoids are recognized for their essential role in photosynthesis and photoprotection (Ramel et al., 2012; Domonkos et al., 2013; Niyogi and Truong, 2013). Carotenoids also are precursors for abscisic acid (ABA) and strigolactone synthesis (Crozier, 2000; Gomez-Roldan et al., 2008), which has been demonstrated to be involved in responses to abiotic stresses, including high temperature and drought (Li et al., 2008a; Li et al., 2008b; Park et al., 2016) and shoot branching and plant signaling, respectively (Yoneyama et al., 2018). In mammals, carotenoids constitute an essential group of nutrients, which function as vitamin A precursors, stimulating immune system and antioxidant activity (Hughes, 1999; Cazzonelli and Pogson, 2010). Studies have demonstrated that in humans (Seddon et al., 1994; Snodderly, 1995; Fiedor and Burda, 2014) carotenoids have an important antioxidant and anticancer activity, as well as protector agents against macular degeneration of elderly people (Beatty et al., 2000). Because of their multiple functions, efforts have been made to elucidate carotenoids synthesis and storage in different plant species, and work has been made to fortify staple crops for carotenoid consumption (Zhu et al., 2018; Tian et al., 2019). Carotenoid accumulation in chromoplasts is the result of three independent processes, carotenoid biosynthesis, degradation, and stable storage, which occur at different developmental plant stages (Li and Yuan, 2013; Nisar et al., 2015). Specific transcription factors establish a tight and precise control of gene expression along carotenoid pathway, allowing biosynthesis and catabolism in accordance with specific organ requirements (Young and Pallett, 2017). In the present article, the focus is made on the role of Orange protein as a regulator of carotenoid accumulation in different plant species and its interaction with phytoene synthase, which has been named a key enzyme in the carotenoid pathway, with special emphasis on efforts to manipulate the *Orange* gene to stimulate carotenoid accumulation. Strategies to achieve this goal are proposed, considering the recent advances in plant genome editing.

## Presence of *Orange gene* in Higher Plants

Recognized as a dominant spontaneous mutation in *Brassica oleracea*, which confers orange color to the influorescence, *Orange* gene has proven to play an important role in carotenoid accumulation by activating chromoplast differentiation in nongreen tissues (Lu et al., 2006). In cauliflower, the mutant allele consists of a large retrotransposon insertion, with homozygous mutants displaying a dwarf phenotype, which include longer petiole, small curds, and late flowering (Li et al., 2001; Lu et al., 2006). *Orange* gene (*OR*) encodes for a protein belonging to the DnaJ cysteine-rich zinc finger protein domain, which is a highly conserved sequence among diverse plant species (Lu et al., 2006; Tzuri et al., 2015; Pulido and Leister, 2018), had positioned Orange protein in the nucleus (Zhou et al., 2011; Kim et al., 2013; Sun et al., 2016), suggesting that interaction with nuclear elements such as transcription factors is required to initiate plastid transition and carotenoid accumulation (Sun et al., 2016). Orthologs of cauliflower wild-type *OR* have been identified and have been shown to have a positive effect on carotenoid increase exhibiting important responses to environmental stress (Kim et al., 2013; Tzuri et al., 2015; Yuan et al., 2015a; Yuan et al., 2015b). In experiments conducted in *Arabidopsis* and sweetpotato, expression of orthologs (*IbOR* and *AtOR*, respectively), were able to induce carotenoid accumulation in different tissues, such as sweetpotato plants, rice callus, and rice plants (Kim et al., 2013; Bai et al., 2016), increasing tolerance to NaCl stress, by moderating oxidation states in transgenic calli. During the postharvest period in potato, expression of *Orange* stimulates continuous accumulation and stable storage of carotenoids (Li et al., 2012), opening the possibilities to decrease losses during potato storage. In melon, a single mutation of a residue in *Orange* gene, the golden SNP, produces a change from green to orange flesh in fruits, with carotenoid accumulation from day 30 after anthesis, with no further effects on phenotype (Tzuri et al., 2015), increasing variability in fruits. Isolation and expression of mutated *Sorghum bicolor Orange* gene in *Arabidopsis* led to an increase in carotenoid accumulation in calli (Yuan et al., 2015a), with no additional effects on carotenoid metabolic gene expression. In mutant *B. oleracea*, *Orange* has been associated also with plant growth and development (Zhou et al., 2011). Analysis of the interaction of BoOr and cauliflower eukaryotic release factor 1 protein (eRF1) revealed an antagonist effect, with an increase in the length of the petiole because of suppression of *BoeRF1* family genes when BoOr was expressed (Zhou et al., 2011).

## Orange Protein Mode of Action and Carotenoid Regulation

Regulation of key biosynthetic genes is a major determinant for carotenoid content (Cong et al., 2009; Vallabhaneni and Wurtzel, 2009; da Silva Messias et al., 2014; Yuan et al., 2015b). In *Arabidopsis*, AtOr protein is located in plastids, and its N-terminal region interacts directly with phytoene synthase (PSY) to control carotenoid biosynthesis posttranscriptionally (Zhou et al., 2015). Expression of carotenoid biosynthetic genes in *B. oleracea* was not changed by the *Orange B. oleracea* (*BoOR*) dominant mutation, with normal levels of PSY expressed during development (Li et al., 2001). Natural allelic variations of the *Orange* gene in melon fruits, *CmOR*^His^ and *CmOR*^Arg^, similarly enhanced carotenoid biosynthesis by posttranscriptionally regulating PSY proteins amounts (Tzuri et al., 2015). In sweetpotato, IbPSY interacts with the IbOr-N fragment, which contains the N-terminal region and the transmembrane domain (Park et al., 2016), which have been reported to mediate protein–protein interaction (Zhou et al., 2015). Later studies had proven also that sweetpotato and melon orange protein (IbOr and CmOr) interact with PSY providing holdase activity (Park et al., 2016), protecting PSY under heat and oxidative stress conditions, keeping the protein structure and preventing aggregation by protein association. It has been also hypothesized that Orange protein function is to keep the levels and efficiency of photosystem II and chlorophyll under high-temperature conditions (Kim et al., 2013; Wang et al., 2015; Cho et al., 2016; Park et al., 2016). Further studies have concluded that the active form of PSY is given by the formation of the membrane-complex PSY-Orange, which is responsible for carotenogenesis (Welsch et al., 2018), while nonassociated, misfolded PSY is subject of degradation by Clp proteases; thus, Orange protein constitutes a key enzyme for the regulation of carotenoid synthesis (Chayut et al., 2017; Welsch et al., 2018).

The creation of a metabolic sink, via promoting the formation of carotenoid-sequestering structures, can additionally modulate carotenoid content (Li and Van Eck, 2007; Sun et al., 2018) and is also function of the *Orange* gene (Chayut et al., 2017). In *B. oleracea*, this mutation induces accumulation of high levels of β-carotene by elevated biogenesis of chromoplasts (Li and Yuan, 2013) up-regulating the plastid formation/translocation factor gene (Lu et al., 2006). Because of the evidence of the formation of the active membrane complex PSY-Orange and its function on carotenoid formation, chromoplast differentiation in presence of the complex might be triggered by accumulation of carotenoids above the threshold required to stimulate plastid differentiation (Bai et al., 2014; Welsch et al., 2018). The influence of Orange affects not only nongreen tissue. Experiments in tomato showed that expression of *AtOR^His^* also increased the content especially of β-carotene in tomato flowers and fruits (Yazdani et al., 2018), organs with a high carotenoid content. In absence of functional Orange protein (Chayut et al., 2017), chromoplast formation was inhibited as early as 30 days after anthesis, and the effect was maintained through fruit ripening, illustrating the temporal accumulation of β-carotene and lutein. In addition, *Orange* gene regulates plastid replications, so each cell contains a single chromoplast (Paolillo et al., 2004), densely packed with carotenoids.

Studies have demonstrated that total carotenoid content is also regulated by carotenoid degradation (Vallabhaneni and Wurtzel, 2009; Gayen et al., 2015). An inverse effect in *Arabidopsis* carotenoid content is given when there is a loss of function of the carotenoid cleavage dioxygenase 1 (CCD1) resulting in an increase in carotenoids (Auldridge et al., 2006). In later studies, it was well established that an increase in the number of CCD1 copies, leading to transcript accumulation during grain development, negatively correlated with maize seed carotenoid content (Vallabhaneni et al., 2010; da Silva Messias et al., 2014). Endosperm carotenoid content in maize varies widely from yellow to white, showing the highest CCD1 expression level in the latter, because of the action of multiple copies of CCD1 exhibiting carotenoid degrading activity, which are encoded by white cap locus (Tan et al., 2017). Likewise, loss of function in CCD4 induces carotenoid accumulation during grain maturation, resulting in an increase of eightfold in β-carotene content in *Arabidopsis* (da Silva Messias et al., 2014). In sweetpotato overexpressing *IbOR*, it was found that 9-*cis*-epoxycarotenoid dioxygenases, CCD1, and CCD4 were highly expressed and that Orange interacts specifically with CCD4, suggesting that Orange has an important role regulating carotenoid homeostasis by preventing degradation (Park et al., 2015). In melon, Orange-mediated β-carotene accumulation has been demonstrated to be the result of downstream metabolism inhibition (hydroxylation or degradation) (Chayut et al., 2017). This mechanism is species-dependent (Tzuri et al., 2015); previous studies have shown that overexpression of *CmOR*^His^ or *AtOR*^His^ promotes β-carotene accumulation, along with significant amounts of lutein, in nongreen tissue of *Arabidopsis* (Tzuri et al., 2015). *CmOR* expression was positively correlated with chromoplast formation, having an important effect on the stability and metabolism of β-carotene, contributing with no effect on the carotenoid pathway reactions upstream of β-carotene formation (Chayut et al., 2015; Tzuri et al., 2015). Only expression of *CmOR* ^His^ prevented further degradation of carotenoid, resulting in accumulation in chromoplasts(Tzuri et al., 2015).

## Manipulating *Orange gene* Toward Carotenoid Accumulation

Low levels of carotenoid in major crops have led to efforts to increase its contents through the development of carotenoid-enriched staple food (Fraser and Bramley, 2004). By altering the key biosynthesis and degradation gene levels, several crops with high carotenoid content have been developed (Fraser et al., 2002; Ducreux et al., 2004; Paine et al., 2005; Diretto et al., 2007a; Naqvi et al., 2009; Welsch et al., 2010). Overexpression of carotenoid *PSY* gene from bacteria or plants resulted in significant increases in total carotenoid levels in tomato fruits (Fraser et al., 2002), potato tubers (Ducreux et al., 2004), and canola seeds (Shewmaker et al., 1999). However, overexpression of *PSY* in canola and *Arabidopsis* led to a delay in germination because of an undesired collateral effect such as an increase in carotenoid-derived ABA (Shewmaker et al., 1999; Lindgren et al., 2003). In transgenic tomatoes, accumulation of *PSY-1* resulted in limited growth due to insufficient gibberellin synthesis, as well as altered fruit pigmentation (Fray et al., 1995). Therefore, a different approach was followed in later experiments, by combining expression of different genes belonging to the carotenoid pathway, which led to substantial β-carotene increases in rice (Ye et al., 2000; Paine et al., 2005; Tian et al., 2019) and potato (Diretto et al., 2007a; Diretto et al., 2007b). A variation of the method was proven in maize; by selecting those favorable alleles that had been verified to alter the metabolic pathway toward β-carotene gave as an end result orange maize seeds with extended β-carotene content (Harjes et al., 2008; Yan et al., 2010; Zhu et al., 2018). However, in some cases, the increased flux into carotenoid biosynthesis can alter or reduce flux to other important pathways, leading to undesired changes (Cazzonelli and Pogson, 2010).

The manipulation of *Orange* genes (**Table 1**) provides an alternative and complementary strategy to increase carotenoid levels by increasing chromoplast differentiation (Li and Van Eck, 2007) and stabilizing PSY (Welsch et al., 2018). The expression of mutant *BoOR* in potato tubers gave an increase in carotenoid content (Lopez et al., 2008) contributing to potato tuber storage during postharvest conditions (Li et al., 2012). The sweetpotato ortholog, *IbOR*, induced carotenoid accumulation in sweetpotato callus, likely as a result of the formation of PSY-Orange complex, favoring carotenoid biosynthesis (Kim et al., 2013; Welsch et al., 2018). Carotenoid isomerase, lycopene cyclase B, and plastid fusion/translocation factor were highly expressed in the *IbOR* transgenic calli, resulting in an increase of 12-fold in total carotenoid content (Kim et al., 2013).

**Table 1:** Effect of *Orange* gene on carotenoid expression in higher plants.

Species gene donor	Host/organ	Mutation	Function	Reference
*Brassica oleracea*	*Solanum tuberosum*/tubers	*BoOR*^Mut^	Carotenoid accumulation and chromoplast formation	(Lopez et al., 2008)
*B. oleracea*	*S. tuberosum*/tubers	*BoOR*^Mut^	Stable carotenoid accumulation during postharvest	(Li et al., 2012)
*Ipomoea batatas*	*I. batatas*/callus	*IbOR-Ins*	Carotenoid accumulation Salt stress tolerance	(Kim et al., 2013)
*Arabidopsis thaliana*	*Oryza sativa*/calli	*AtOR*^His^	Carotenoid accumulation	(Bai et al., 2014)
*I. batatas*	*Medicago sativa*/leaves	*IbOR-Ins*	Carotenoid accumulation Abiotic stress tolerance	(Wang et al., 2015)
*Sorghum bicolor*	*A. thaliana*/callus	*SbOR^His^*	Carotenoid accumulation	(Yuan et al., 2015a)
*Cucumis melon*	*A. thaliana*/callus	*CmOR^His^*	Carotenoid accumulation	(Tzuri et al., 2015)
*A. thaliana*	*O. sativa*/seeds	*AtOR*^His^	Carotenoid accumulation	(Bai et al., 2016)
*I. batatas*	*S. tuberosum*/tubers	*IbOR-Ins*	Tuber yield Abiotic stress tolerance	(Cho et al., 2016)
*I. batatas*	*A. thaliana*/callus	*IbOr-Ins*	Abiotic stress tolerance	(Park et al., 2016)
*A. thaliana*	*Zea mays*/seeds	*AtOR*^His^	Carotenoid accumulation	(Berman et al., 2017)
*A. thaliana*	*Solanum licopersicum*/fruit	*AtOR*^His^	Carotenoid accumulation	(Yazdani et al., 2018)
*M. sativa*	*Nicotiana benthamiana*/leaves	*MsOr*	Carotenoid accumulation/environmental stress tolerance	(Wang et al., 2018)

By mimicking the naturally occurring mutation in melon, where a single Arg to His substitution in *Orange* gene is responsible for the orange-fleshed phenotype (Tzuri et al., 2015), a change was introduced to *Arabidopsis thaliana* to generate *AtOR* ^His^ mutants, which were able to accumulate carotenoids in calli (Yuan et al., 2015a). From this finding, it was proven that the His mutation in *Orange* gene might change or create a new active center or binding site, leading to accumulation of carotenoids (Yuan et al., 2015a), broadening the possibilities of generating crops with enhanced carotenoid content.

The expression of *AtOR* also induced carotenoid accumulation in rice callus (Bai et al., 2014) and seeds (Bai et al., 2016). In this latter case, expression of three genes involved in carotenoid synthesis was achieved. *Arabidopsis thaliana Orange* (*AtOR*) gene, which was shown to successfully sequester carotenoids in rice callus, was expressed under control of an specific endosperm promoter, along with two carotenogenic genes, *Zea mays* phytoene synthase1 (*ZmPSY*1) and *Pantoea ananatis* phytoene desaturase (*PaCRTI*) (Bai et al., 2014). In the experiment, combined expression of *ZmPSY*1, *PaCRTI*, and *AtOR* enhanced carotenoid increase through the creation of a metabolic sink, which resulted in the positive regulation of several endogenous carotenogenic genes (Bai et al., 2014). Measurement of total carotenoid content when all three genes were expressed (*AtOR*, *ZmPSY*1, and *PaCRTI*) resulted in an increase of at least twofold of carotenoid levels, with prevalence of higher levels of β‐carotene and α‐carotene (Bai et al., 2014). In a different experiment, the level of endogenous *Oryza sativa* lycopene cyclase-β (*OsLCY-*β) increased in rice lines expressing *AtOR*, suggesting endogenous *OsLCY-*β is sufficient for enhanced β‐carotene synthesis and accumulation in lines overexpressing 1-deoxy--xylulose 5-phosphate synthase as might be expected given the greater presence throughout the entire pathway (Bai et al., 2016). This situation can be explained by the level of endogenous *OsLCY-*ε mRNA, which was positively regulated in those lines expressing *AtOR*, resulting in a fivefold increase in α‐carotene accumulation in seeds (Bai et al., 2016).

In a later experiment, expression of *AtOR* under the control of an endosperm-specific wheat low-molecular-weight glutenin promoter resulted in an elevated carotenoid content in white corn (Berman et al., 2017), by promoting the formation of carotenoid-sequestering plastoglobuli when the carotenoid pool was limited, but it had no effect when carotenoid levels where abundant, in which case carotenoid storage structures can be induced even in the absence of *AtOR* (Berman et al., 2017). On the other hand, in transgenic tomato, overexpression of *AtOR*^His^ mutant enhanced the accumulation of carotenoids in nongreen tissue by promoting chloroplast to chromoplast conversion and induced carotenoid accumulation at early fruit developmental stages (Yazdani et al., 2018), when using *Cauliflower mosaic virus 35S* as promoter; therefore, further research is needed to elucidate the role of promoters in Orange gene expression and carotenoid content. In addition to these findings, it was found that *AtOR*^His^ mutant promoted early flowering, fruit set, and seed production, three agronomically important characters for increasing fruit yield as well as to develop varieties adapted to harsh conditions such as chill injury or short summer season (Yazdani et al., 2018),

Besides to their role in carotenoid accumulation, *OR* genes have been shown to enhance tolerance to environmental stress. In sweetpotato, overexpression of *IbOR* enhanced tolerance to heat and oxidative stress (Park et al., 2016; Kang et al., 2017). Transgenic alfalfa and potato overexpressing *IbOR* showed a remarkable resistance to a variety of abiotic stresses, such as drought, salinity, and heat (Wang et al., 2015; Cho et al., 2016). Transcriptome analysis at early fruit development in tomato showed that eight common genes at *AtOR* ^His^ were heat shock proteins, supporting the hypothesis that *Orange* might play an important role during heat stress (Yazdani et al., 2018); however, because of the lack of J domain on the protein, Orange protein mode of action is proposed to be by interaction with the substrate by the Zinc finger and C-terminal domain (Pulido and Leister, 2018), and therefore, expression of Orange proteins can be linked to photosynthesis maintenance, by increase in carotenoid levels by enhancing sink strength in storage tissues leads to stress tolerance in plants (Wang et al., 2015; Cho et al., 2016; Kim et al., 2018; Wang et al., 2018).

## Proposed Strategies to Modify *Orange gene*

The advent of the highly efficient CRISPR/Cas9 system for eukaryote gene editing (Jinek et al., 2012; Cong et al., 2013), with rice being the first edited plant genome (Zhang et al., 2014; Zhang et al., 2016), opened the possibility to mutate target genes in crop species (Shan et al., 2013). Utilization of CRISPR/Cas9 complex to generate double-stranded breaks under the specific guidance of a single guide RNA provides the opportunity to mutate the gene of interest to produce desire effect on the progeny (Scheben et al., 2017). Originally designed to rely on homologous recombination, or more commonly, on nonhomologous end-joining, to lead the introduction of mutations such as small insertions or deletions in the targeted DNA molecules (Jinek et al., 2012; Cong et al., 2013), the increasing need to incorporate specific mutations for genome editing in plants has headed to the development of novel techniques, such as “base editor” (Hua et al., 2018; Kang et al., 2018; Li et al., 2018; Yan et al., 2018; Qin et al., 2019). Successful edition of GhCLA and GhPEBP by a base editor consisting of a cytidine deamidase domain fused with nCas9 and UGI resulted in a 57% of base-editing efficiency with no detectable unwanted mutations on the progeny (Qin et al., 2019). This development offers advantageous possibilities to mutate Orange to increase carotenoid accumulation in crop species, taking advantage of His/Ala mutation (Yuan et al., 2015a; Yuan et al., 2015b) at conserved Arginine residue, and also to explore the function of residues at Zinc finger domain and the rationale behind PSY stabilization.

Nevertheless, optimization of CRISPR technology is also needed for each species to accommodate the tissue and transformation delivery method (Char et al., 2017). Today, two major transformation methods are utilized for DNA delivery, *Agrobacterium tumefaciens* or biolistic transformation (gene-gun), with both methods being effective in transforming different plant species, with Agrobacterium based-method, being more popular and efficient for the insertion of low numbers of transgenes (Khatodia et al., 2016; Char et al., 2017). Lately, the development of new transformation techniques based on the utilization of functionalized nanoparticles to deliver DNA has been proven with success in species such as cotton, sunflower, and lily (Zhao et al., 2017). With the aid of magnetic fields, nanoparticles can efficiently deliver CRISPR vectors through pores present in the pollen grains, producing transformed pollen, which is then used to pollinate emasculated flowers, resulting in transformed seeds (Zhao et al., 2017). This increases transformation efficiency and bypasses the procedure of tissue culture to generate plants from transformed seeds within a short period of time, allowing the introduction of functional alleles of Orange into staple crops, contributing to existing breeding programs worldwide.

## Conclusion and Future Prospects

To date, despite the evidence that supports Orange protein playing an important role in plant growth and development, only a few species have characterized the gene, including cauliflower, *Arabidopsis*, melon, sweetpotato, and alfalfa. *Orange* represents an exceptional class of regulatory genes that mediate carotenoid accumulation, being highly conserved among different species, with critical functions, such as maintaining carotenoid homeostasis and acting directly under environmental stress, controlling carotenoid biosynthesis, and stabilizing photosynthesis.

Further examination of the *OR* effects in different species and its diversity among them will help to elucidate fully the functional role of this gene, allowing its manipulation to enhance β‐carotene levels. With the advent of genome editing techniques, which can direct specific point mutations in the genome, there are certain possibilities to modify the *Orange* gene sequence to increase carotenoid accumulation in staple crop species such as wheat, corn, canola, and rice by nontransgenic approach. There is enough evidence to support that *Orange* genes could be used to cope with the effects of climate change; therefore, its manipulation could be suitable to use in strategic crops, widely distributed, to develop varieties with increased tolerance to heat and water stress, in addition to improved nutritional qualities associated with higher levels of carotenoids.

## Author Contributions

CO developed the idea and wrote the manuscript.

## Funding

The author thanks financial support from CONICYT REGIONAL/ GORE ARAUCANIA/ CGNA R16A10001.

## Conflict of Interest

The author declares that the research was conducted in the absence of any commercial or financial relationships that could be construed as a potential conflict of interest.
